# Searching for Promoters to Drive Stable and Long-Term Transgene Expression in Fibroblasts for Syngeneic Mouse Tumor Models

**DOI:** 10.3390/ijms21176098

**Published:** 2020-08-24

**Authors:** Dina V. Antonova, Irina V. Alekseenko, Anastasiia K. Siniushina, Alexey I. Kuzmich, Victor V. Pleshkan

**Affiliations:** 1 Department of Genomics and Postgenomic Technologies, Gene Immunooncotherapy Group, Shemyakin-Ovchinnikov Institute of Bioorganic Chemistry of the Russian Academy of Sciences, 117997 Moscow, Russia; tyulkina.dina@mail.ru (D.V.A.); irina.alekseenko@mail.ru (I.V.A.); nastyasiniushina@gmail.com (A.K.S.); akrubik@gmail.com (A.I.K.); 2 Gene Oncotherapy Sector, Institute of Molecular Genetics, Russian Academy of Sciences, 123182 Moscow, Russia; 3 Laboratory of Epigenetics, National Medical Research Center for Obstetrics, Gynecology and Perinatology Named after Academician V.I. Kulakov of the Ministry of Healthcare of Russian Federation, 117997 Moscow, Russia

**Keywords:** promoter, tumor microenvironment, fibroblasts, mouse model, cell proliferation

## Abstract

Tumor is a complex system of interactions between cancer cells and other cells of the tumor microenvironment. The cancer-associated fibroblasts (CAFs) of the tumor microenvironment remain in close contact with the cancer cells and play an important role in cancer progression. Genetically, CAFs are more stable than cancer cells, making them an attractive target for genetic modification in gene therapy. However, the efficiency of various promoters for transgene expression in fibroblasts is scarcely studied. We performed a comparative analysis of transgene long-term expression under the control of strong cytomegalovirus promoter (pCMV), constitutive cell promoter of the *PCNA* gene (pPCNA), and the potentially fibroblast-specific promoter of the *IGFBP2* gene (pIGFBP2). In vitro expression of the transgene under the control of pCMV in fibroblasts was decreased soon after transduction, whereas the expression was more stable under the control of pIGFBP2 and pPCNA. The efficiency of transgene expression was higher under pPCNA than that under pIGFBP2. Additionally, in a mouse model, pPCNA provided more stable and increased transgene expression in fibroblasts as compared to that under pCMV. We conclude that PCNA promoter is the most efficient for long-term expression of transgenes in fibroblasts both in vitro and in vivo.

## 1. Introduction

Cancer cells actively interact with each other and with the tumor microenvironment (TME), thus strongly enhancing the complexity of cancer [1,2,3].

It has been suggested that one of the main causes of development of malignant cancer phenotype is the interaction of cancer cells with a major component of TME, cancer-associated fibroblasts (CAFs) [4,5,6,7].

In contrast to genetically unstable cancer cells, CAFs, which are in close contact with them, are significantly more stable [8,9,10,11]. This makes CAFs an attractive target for stable genetic modification in gene therapy [9,12].

Gene therapy for malignant tumors requires a prolonged functional activity of genetic constructs transduced into the cells. The behavior of transgenic constructs has been most studied in cancer cells. In these studies, apart from strong non-specific promoters such as CMV (cytomegalovirus immediate-early promoter), various tumor-specific promoters (e.g., survivin promoter) and chimeric promoters with a wide expression profile in various cancer cells have also been used [13,14,15]. However, the problem of the prolonged functioning of promoters in tumor fibroblasts requires further studies.

Cells use various mechanisms to silence the transgene integrated into their genome. Most commonly, the transgene inserted into the genome as part of a retroviral vector undergoes promoter-dependent silencing through methylation of the transgene promoter [16,17]. For example, in hepatocellular carcinoma transduced with lentiviruses containing *EGFP* gene under the control of CMV promoter, the number of cells expressing *EGFP* was decreased by ~30% on day 15 and by 70% on day 30 after transduction. When the transgene promoter pPGK (the promoter of phosphoglycerate kinase) was used, the gene expression persisted for up to 2 months after the transduction [18]. Apart from DNA methylation, the transgene can be silenced through histone modification [19].

The behavior of genetic constructs in TME has been less studied. To ensure the expression of transgenes in fibroblasts, non-specific viral promoters, such as the strong CMV promoter, or promoters specific for fibroblasts, e.g., the promoter of *FSP1* (the gene of fibroblast specific protein-1) [20,21,22], or fibroblast-specific regulatory elements subcloned from the upstream region of the mouse *Col1a2* gene [23,24,25] are used. Alternatively, constitutive human EF1a or mouse pPGK promoters are used [26]. However, sometimes the transcription from promoter EF1a becomes an order of magnitude lower a few days after transfection [27]. The pPGK promoter ensures a low level of expression, the duration of which is unknown [28].

In this study, we used genetic constructs under control of the promoters—strong cytomegalovirus promoter (pCMV), constitutive cell promoter of the *PCNA* gene (pPCNA), and the potentially fibroblast-specific promoter of the *IGFBP2* gene (pIGFBP2) for genetic modification of fibroblasts. A high level of expression was observed with the strong CMV promoter, although it may be silenced during prolonged culturing of the transduced cells. The constitutive PCNA promoter is a potentially universal promoter for gene therapeutic constructs [29]. We used the insulin-like growth factor binding protein-2 (IGFBP2) promoter, as it is a potentially fibroblast-specific promoter; however, it is comparatively less active. The pIGFBP2 promoter was taken as a weak but potentially capable option to provide long-term expression of the transgene in the CAFs. We analyzed the ability of these promoters to sustain efficient and long-term transgene expression in primary culture of cancer-associated fibroblasts and fibroblast cell lines in vitro. To this end, we obtained a lentiviral vector containing these promoters controlling expression of a bicistronic construct consisting of a suicide gene *HSVtk* and a reporter gene *GFP* connected via a sequence of P2A peptide. Cells transduced by lentiviruses were analyzed for the transgene expression (by qPCR, FACS, and MTS-test) after 1 and 4–5 weeks post transduction. In addition, we evaluated the ability of pCMV and pPCNA promoters to provide transgene expression in fibroblasts in vivo in a mice tumor model. To do this, lentiviruses were created that contained the reporter genes of green fluorescent protein and firefly luciferase (*Luc2*) under the control of the pCMV or the pPCNA promoter, linked via a P2A sequence. BALB/3T3 mice fibroblasts were transduced with the obtained lentiviruses and then inoculated to mice as part of a co-culture with CT26.WT cancer cells. Two weeks after co-culture inoculation, tumors were collected, and the number of GFP+ cells and the relative luciferase activity were determined. Our results indicate that the type of promoter employed can be critical in the establishment of genetically modified fibroblasts.

## 2. Results

### 2.1. Obtaining Human and Mouse Fibroblasts with Stable Expression of the HSVtk-P2A-GFP Cassette under the Control of Various Promoters

**Figure 1:**
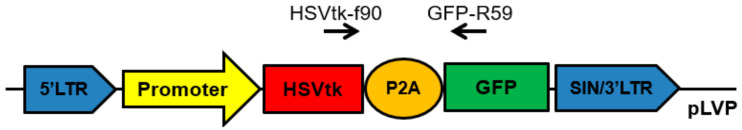
Scheme of the obtained lentiviral constructs developed for in vitro studies. Promoter: pCMV, pPCNA, or pIGFBP2. HSVtk: herpes simplex virus thymidine kinase gene. GFP: green fluorescent protein (CopGFP) gene used as a reporter gene. P2A: the DNA-sequence of P2A peptide of the porcine teschovirus-1. HSVtk-f90 and GFP-R59: a universal pair of primers used to determine the transcription level of the *HSVtk-GFP* transgene.

For in vitro studies, we prepared lentiviral constructs containing the suicide gene *HSVtk* and reporter gene *GFP* linked together by the DNA-sequence of P2A peptide, which allows researchers to obtain products of both genes in a ratio of approximately 1:1 (Figure 1) [30]. The constructs differed only in promoters.

We tested three promoters: strong, universal, and constitutive pCMV, strong and potentially universal promoter of mammalian cells, pPCNA, and a 640-bp region of pIGFBP2 located upstream of the first exon and having a weak but potentially fibroblast-specific promoter activity. The promoter region within pIGFBP2 is GC-rich, does not contain TATA and CAAT sequences, and has its own transcription start site located within a region of 113–115 bp from the translation initiation site ATG [31]. Using the constructs shown in Figure 1, we obtained lentiviruses for transduction of the primary culture of human fibroblasts (IVP-15TS) and cell lines of mouse fibroblasts (NIH/3T3, L-929).

We measured the mRNA levels of the *HSVtk-GFP* transgene and fluorescence intensity of the reporter GFP protein in the stably transduced fibroblasts. We also determined the number of GFP+ cells and tested the cytotoxicity of HSVtk in the presence of ganciclovir. To evaluate any changes in the activity of the promoters used during prolonged culturing in vitro, we analyzed their activity twice, 1 and 4–5 weeks post transduction. For mouse fibroblast cell lines NIH/3T3 and L-929, the analysis was performed 5 weeks after their transduction. For the primary culture of human fibroblasts, the analysis was performed 4 weeks after their transduction because their rate of cell proliferation was significantly decreased by the fourth week of transduction. The experiment was repeated twice for NIH/3T3 and L-929 fibroblasts, and the profiles of observable changes were similar.

### 2.2. Gene Copy Number Analysis

To exclude a possible undesirable effect of unequal viral integration, the quantity of proviral DNA was measured by quantitative real-time PCR (qPCR) after 1 and 5 weeks of transduction. There were no principal changes in the number of integrations during cell culture, i.e., the changes observed during cell culture were not caused by deletions or amplifications of the transgene (Figure S1).

### 2.3. Determination of Relative Transcription Level of the HSVtk-GFP Transgene

**Figure 2:**
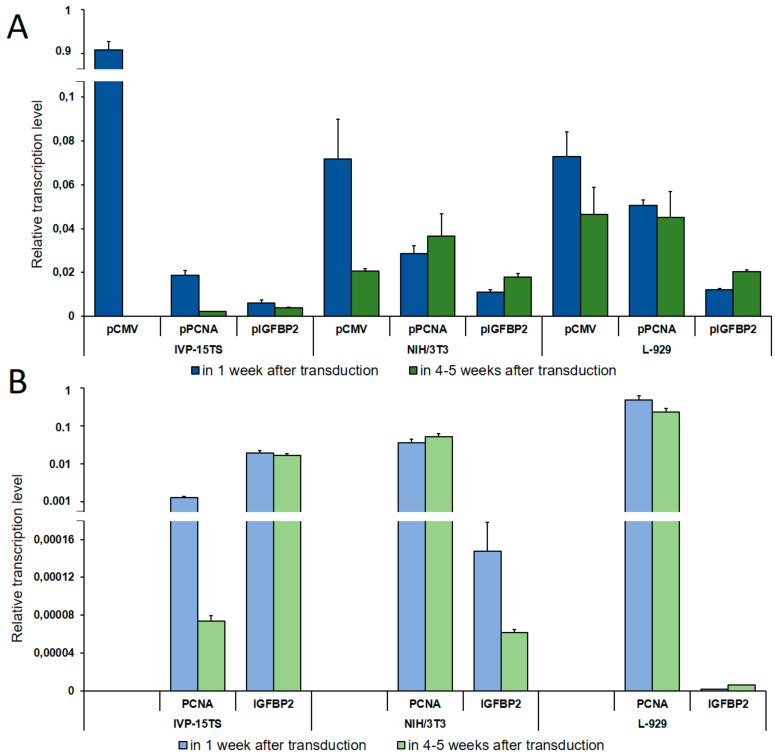
Determination of Relative Transcription Level. (**A**) Relative transcription level of the transgene under the control of pCMV, pPCNA, and pIGFBP2 promoters as determined by qPCR analysis. (**B**) Relative transcription level of the *PCNA* and the *IGFBP2* genes. The names of promoters or genes are indicated below the abscissa axis. The measurements were performed in three independent replicate experiments for each sample and are represented as mean ± s.e.m.

The transcription level of the *HSVtk*-*GFP* transgene was determined by qPCR using a universal pair of primers (Figure 1). In the cell lines containing the transgene under the control of pPCNA or pIGFBP2, we also measured the transcription levels of the *PCNA* or *IGFBP2* genes, respectively, to compare the activity of pPCNA or pIGFBP2 controlling the transgene expression with that of the *PCNA* and the *IGFBP2* genes. The values obtained were normalized to the geometric mean of transcription levels of the *18S RNA*, *GPI*, and *EEF1A1* genes for the primary culture of human fibroblasts and to those of the *m18S RNA*, *Psmb7*, and *Rab1b* genes for mouse fibroblast cell lines (Figure 2).

As seen in Figure 2, on increasing the culture time of IVP-15TS, the transcription level of the transgene under the control of pCMV or pPCNA decreased sharply. This decrease was accompanied by the decrease in the transcription level of *PCNA* (Figure 2B). In contrast to pCMV and pPCNA, the transcription level of the transgene under the control of pIGFBP2 was markedly lower at the beginning of the experiment, and changes in the transcription level during the experiment were insignificant, such that, by the fourth week of culture, the transcription level under pIGFBP2 was higher than that under pCMV or pPCNA. The transcription level of *IGFBP2* also changed insignificantly. In mouse fibroblast cell lines, the transcription level of the transgene during long-term cell culture decreased only under pCMV. In IVP15-TS, the transcription level of the transgene under pPCNA and that of *PCNA* changed insignificantly. In NIH/3T3 cells, the expression of the transgene under the control of pPCNA and that of the *PCNA* gene increased in the course of the experiment. This was probably due to spontaneous transformation and malignization of the embryonic fibroblasts [32], which may have been accompanied by an enhanced expression of the *PCNA* gene [33]. The expression of transgene under pIGFBP2 in mouse fibroblast cell lines L-929 and NIH/3T3 also increased.

During cell cultivation, transcription of the transgene from the pPCNA and the pIGFBP2 promoters in the NIH/3T3 and the L-929 fibroblasts was the most stable, while the relative number of transcripts from the pPCNA promoter was three to five times higher than from the pIGFBP2 promoter. Immediately after transduction, the relative transcription level of the transgene from the pCMV promoter was the highest among all promoters in all cell lines. Relative transcription level did not differ in NIH/3T3 and L-929 fibroblasts and was an order of magnitude higher in the primary culture IVP-15TS. However, during long-term cultivation of cells, the decrease in transcription from pCMV was the highest possible, a complete inhibition in IVP-15TS, an order of magnitude decrease for NIH/3T3, and a two-fold decrease for L-929 fibroblasts.

### 2.4. Evaluation of GFP Expression

**Figure 3:**
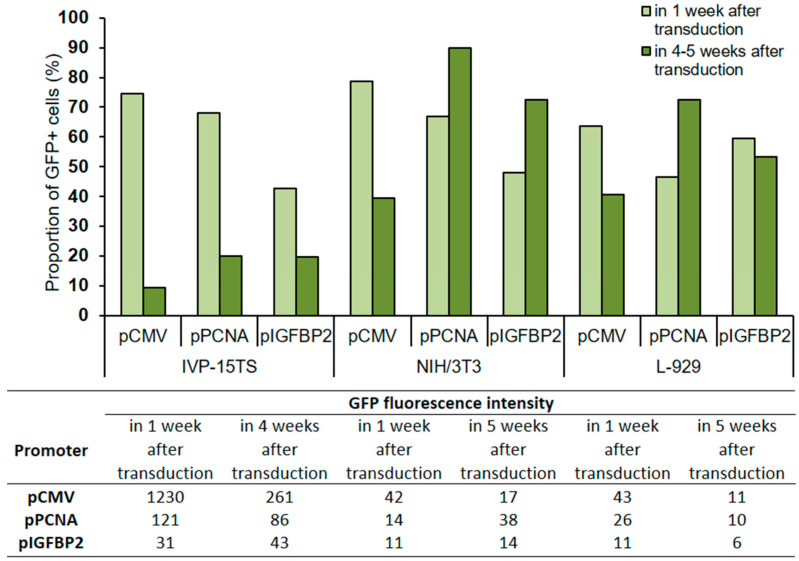
Proportion of GFP+ cells and GFP fluorescence intensity in transduced cells after 1 and 4–5 weeks of culture post transduction. The total amount of cells in the analysis was taken for 100%. The analysis was performed 4 weeks after transduction for the primary culture (IVP15-TS) and 5 weeks after transduction for linear fibroblasts NIH/3T3 and L-929. The names of promoters or cell lines are indicated below the abscissa axis and correspond both to the graph and to the table.

The expression level of the GFP protein in transduced cells was evaluated by determining the number of GFP+ cells and GFP fluorescence intensity in the transduced cells after 1 and 4–5 weeks of transduction (Figure 3).

In all transduced cells IVP-15TS, the proportion of GFP+ cells decreased with prolonged cultivation regardless of the type of promoter used, but the greatest decrease was under pCMV. In cell lines NIH/3T3 and L-929, a decrease in the number of GFP+ cells was observed only under pCMV. The GFP fluorescence intensity was higher under pCMV than that under pPCNA or pIGFBP2.

### 2.5. Toxicity of HSVtk in the Presence of Ganciclovir

In the lentiviral constructs, the promoters under study also controlled the expression of the transgene containing the suicide gene *HSVtk*. HSVtk phosphorylates a guanosine analogue of ganciclovir (GCV) to GCV-monophosphate, which is further converted by host kinases to GCV-diphosphate and GCV-triphosphate. The resulting GCV-triphosphate is similar to 2′deoxyguanosine triphosphate used for DNA replication and thus gets incorporated into the growing DNA chain. As a result, replication is terminated followed by cell death. We studied the survival rate of transduced cells during growth in a ganciclovir medium to estimate the level of HSVtk production (Table 1).

The cytotoxic effect of the *HSVtk*/GCV system was higher in transduced NIH/3T3 and L-929 cells than in IVP-15TS. The cytotoxic effect of the *HSVtk*/GCV system under the promoters pPCNA and pIGFBP2 was markedly decreased in IVP-15TS during cultivation. Under pCMV, this cytotoxicity decreased in the cell culture of both IVP-15TS and L-929. At first (in 1 week after transduction), the cytotoxicity of the *HSVtk*/GCV system did not depend upon the strength of the promoter in any of the tested cell cultures. The average IC_50_ was about 0.1 μM and ~10 μM in mouse fibroblast cell lines and IVP-15TS, respectively, for all the promoters. Using melanoma cell lines, we earlier showed that the cytotoxic effect of the *HSVtk*/GCV system did not depend on the promoter strength [34].

### 2.6. Cell Proliferation

**Figure 4:**
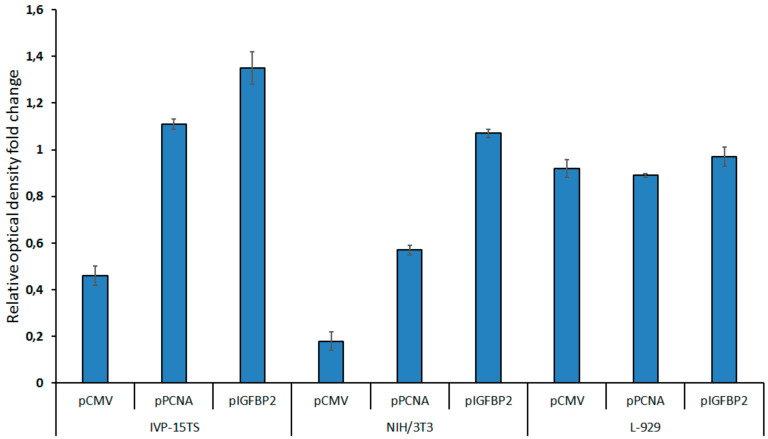
Cell proliferation after 2 weeks of transduction as measured by MTS test. The diagram presents the values of relative optical density of the cells carrying the *HSVtk*-*GFP* transgene under the control of pCMV, pPCNA, or pIGFBP2 promoters. The growth rate of non-transduced control cells was taken as one. The measurements were carried out in three technical replicates for each sample and are represented as mean ± s.e.m.

Proliferation of native (control) and stably transduced cells was determined by MTS analysis (see Figure 4). Cells were seeded into 96-well plates and cultured for 6 days, after which MTS reagent was added and results were analyzed.

In 2 weeks after transduction, the growth of the transduced NIH/3T3 and IVP-15TS fibroblasts containing the transgene under the control of pCMV decreased. The decrease in the rate of cell division rate was directly proportional to the strength of the promoter within the transgene. The stronger the promoter was, the greater was the decrease in rate of the cell division. However, after 4–5 weeks of culture, the rate of cell division was stabilized. The rate of cell division was not much different between the L-929 cells transduced with different transgenes and in 4–5 weeks of the experiment.

Thus, we showed that, in the beginning of the experiment, the expression level of the transgene under the control of pCMV was maximum in all cultures; however, in the course of the experiment, the number of transcripts decreased significantly until no more transcripts were produced (for IVP-15TS fibroblasts). This resulted in considerable decline in the concentration of reporter protein and in the cytotoxicity of the *HSVtk*/GCV system. The expression level of the transgene under the strong pPCNA and the weak but potentially fibroblast-specific pIGFBP2 decreased only in IVP-15TS, which was probably caused by the arrest of cell division during culturing. In NIH/3T3 and L-929 cell lines, the transgene expression under the control of pPCNA and pIGFBP2 remained stable during culturing with the expression level of the transgene being always higher under pPCNA. The cell division of the fibroblasts transformed by transgenes under the control of pCMV was decreased, probably due to the high toxicity of the transgene owing to a high activity of pCMV. Thus, our experiments demonstrated that pPCNA could ensure continuous and long-term expression of the transgene in vitro.

### 2.7. In Vivo Analysis of the pCMV and pPCNA Activity in Fibroblasts

**Figure 5:**
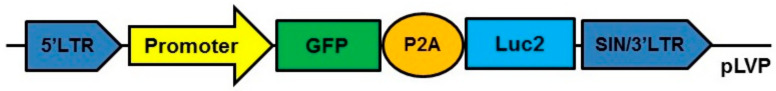
Scheme of the lentiviral constructs used for in vivo experiments. Promoter pCMV or pPCNA. GFP: green fluorescent protein (*EGFP*) gene used as reporter gene. Luc2: *firefly luciferase 2* gene. P2A: the DNA sequence of P2A peptide of the porcine teschovirus-1.

In vitro experiments for pIGFBP2 showed an extremely low level of transgene transcription and the fluorescence signal intensity compared to pCMV and pPCNA. This partially reflects a very weak gene expression controlled by the pIGFBP2 promoter. Our objective was to find a promoter that could provide a relatively high level of transgene expression so that it could be used for modeling in vivo tumor–stroma interactions. Thus, we estimated the ability of pPCNA to ensure the expression of the transgene in fibroblasts modified ex vivo and inoculated subcutaneously into mice along with cancer cells. As a standard, we used pCMV as the promoter for the control of transgene expression. To this end, we prepared lentiviral constructs pCMV-GFP-P2A-Luc2 and pPCNA-GFP-P2A-Luc2 carrying the reporter genes EGFP (enhanced green fluorescent protein) and Luc2 (firefly luciferase 2 gene), respectively, linked by the DNA sequence of P2A peptide and controlled by pCMV or pPCNA (Figure 5).

The constructs prepared were inserted into lentiviruses used for transduction of the BALB/3T3 fibroblasts followed by puromycin selection of the transduced fibroblasts. The use of such fibroblasts was dictated by the fact that these cells can be grafted into Balb/c mice. At the same time, very few CAFs can be isolated from mouse tumors, which rather quickly enter a state of proliferative arrest during in vitro cultivation (which we, in particular, observed for IVP-15TS). This did not allow us to use the CAFs isolated from mice tumors for genetic modification, which requires long-term selection. In 3 weeks after transduction, the proportion of GFP+ fibroblasts carrying the transgene under pCMV was 73%, and that under pPCNA was 89%. The GFP fluorescence intensity in the cells transduced with the cassette containing pPCNA was ~2.5 times higher than that in the cells transduced with the cassette containing pCMV.

Next, the transduced fibroblasts were mixed with CT26.WT cells at a ratio of 3:1 (300 thousand fibroblasts and 100 thousand cancer cells CT26.WT), and the mix was subcutaneously inoculated into mice. We formed three groups of mice: group 1, BALB/3T3-pCMV-GFP-P2A-Luc2 + CT26.WT; group 2, BALB/3T3-pPCNA-GFP-P2A-Luc2 + CT26.WT; and group 3 (control), native BALB/3T3 + CT26.WT. The tumors formed in mice reached the volume of 50–200 mm^3^ within 2 weeks of the inoculation of the co-culture, after which the tumors were resected, fragmented, and treated with collagenase D. The tumor suspension obtained was divided into two parts; the first one was used for measuring the percentage of GFP+ cells and intensity of GFP fluorescence, while the second one was used for measuring the luciferase activity. The results of this analysis are presented in Table 2.

Three of the four tumors obtained from mice after inoculation of the co-culture of CT26.WT and BALB/3T3 cells carrying the transgene under the control of pPCNA contained a significant number of modified fibroblasts (4.08, 3.36, and 1.19%). Two of the three tumors generated in mice after inoculation of the co-culture of CT26.WT and BALB/3T3 cells carrying the transgene under the control of pCMV contained practically no GFP+ fibroblasts (0.03 and 0.11%), while one tumor contained 0.29% GFP+ fibroblasts, which is approximately 1/10 of the number of GFP+ fibroblasts with the transgene under the control of pPCNA. The GFP fluorescence in fibroblasts in the co-culture of BALB/3T3-pCMV-GFP-P2A-Luc2 and CT26.WT cells was approximately 3–4 times lower than that in the co-culture of BALB/3T3-pPCNA-GFP-P2A-Luc2 and CT26.WT cells. The luciferase activity broadly correlated with the number of GFP+ cells in all tumors.

It should be noted that, earlier, we showed that the proportion of Pdgbfrb+ cells, a large part of which might be represented by fibroblasts in the mouse model of colorectal cancer, was no more than 5%. It means that the proportion of modified fibroblasts carrying the transgene under pPCNA in the tumors (1–4%) is close to the native value. Thus, pPCNA is the most efficient promoter for developing genetically modified fibroblasts. In contrast to the widely used pCMV, pPCNA ensures the expression of the transgene for a long time both in vitro and in vivo.

## 3. Discussion 

A tumor is a unified entity of interacting cancer cells around which conditionally normal cells form the tumor microenvironment (TME). TME plays a key role in tumor progression and ensures that the tumor is resistant to traditional methods of treatment. Tumor–stoma crosstalk is now considered an attractive therapeutic target. Creation of new antitumor interactions or destruction of existing pro-tumor interactions in mouse tumor models requires selection of the most promising approaches. Such models can be created by inoculating animals with genetically modified cancer and microenvironment cells. One of the major constituents of TME is CAFs. Genetically modified fibroblasts may be used as CAF precursors for modeling therapeutic interactions in vivo. The use of syngeneic models for these purposes is much simpler than that of immunodeficient animals.

This work was aimed at studying the possible changes in the activity of various promoters for the expression of transgenes inserted in the genome by lentiviral cassettes for long-term culture of transduced or genetically modified fibroblasts in vitro. We also evaluated the possibility of using different promoters for controlling the in vivo transgene expression in fibroblasts. The data obtained may be further used for a rational choice of cancer gene therapy based on modified CAFs.

At present, to express transgenes in fibroblasts, researchers commonly use non-specific viral promoters, fibroblast-specific promoters, e.g., FSP1, or fibroblast-specific regulatory elements subcloned from the upstream region of the mouse *Col1a2* gene [23,24,25]. Since CAFs precursors can be a variety of different cells, the use of a universal promoter rather than a fibroblast-specific promoter may be more justified. Moreover, due to heterogeneity of fibroblasts, the term fibroblast-specific is largely ambiguous. For example, inconsistent results for the activity of the *FSP1* gene promoter in fibroblasts may be due to their heterogeneity [35,36]. Moreover, until recently, no real fibroblast-specific genes were identified, because the so-called “fibroblast-specific genes” were shown to be expressed in both hematopoietic and other cells of an organism [36].

Therefore, in this work, we used three promoters: CMV, the strong universal constitutive promoter of cytomegalovirus; PCNA, potentially universal strong promoter of mammalian cells; and a weak but potentially fibroblast-specific promoter of the *IGFBP2* gene [37] (Antonova DV, to be published elsewhere). Each of these promoters were studied in three different in vitro systems, primary culture of human fibroblasts (IVP-15TS) and mouse fibroblast cell lines (NIH/3T3, L-929), transduced by a lentivirus carrying a transgene composed of the *HSVtk* gene coding part linked to the reporter gene (*GFP*).

We measured three parameters: mRNA level of the transgene, GFP fluorescence intensity, and HSVtk cytotoxicity in the presence of ganciclovir. We followed the changes in these parameters based on fibroblast culture, culturing time, and, in the case of cytotoxicity, on the concentration of ganciclovir.

Our results demonstrated that, in vitro, the pCMV promoter is most inhibited. To determine the possible reasons, we analyzed the quantity of proviral integrations and showed that there are no principal changes in the number of integrations in cell lines during their cultivation. Next, we determined the relative transcription level of the transgene and demonstrated that inhibition of expression in the case of the pCMV promoter occurs precisely at the transcriptional level. The major immediate-early promoter and enhancer of the human cytomegalovirus (hCMV-MIE) is one of the most potent DNA elements driving recombinant gene expression in mammalian cells. Its behavior has been studied rather well [16,17,18,19]. However, the strength of the target gene expression driven by the CMV promoter varies depending on cell types. For example, a CMV promoter driven-green fluorescence protein (GFP) signal was strong in human embryonic kidney cells (293T) and human fibrosarcoma cells (HT1080), while it was weak in fibroblasts (MRC5) and inactivated in mouse embryonic stem cells (D3 and J1) [38]. Thus, our results do not differ from those described in the literature.

It was shown that multi-site methylation of hCMV-MIE is linked to productivity loss in permanently transfected CHO cells lines. In particular, the cytosine located 179 bp upstream of the transcription start site (C-179) is frequently methylated. The single mutation of C-179 to G can significantly stabilize the production of recombinant protein. [39]. Anticipating this possibility, we investigated other expression systems that could allow for more stable effects. The pPCNA seems to be reasonable enough for practical use.

We used a mouse model of colorectal cancer to evaluate the possibility of using the pCMV and the pPCNA promoters for controlling the in vivo transgene expression in fibroblasts. For this, we developed mouse BALB/3T3 fibroblasts stably expressing the reporter genes under the control of pCMV and pPCNA promoters. The co-culture of transduced fibroblasts and CT26.WT cells was inoculated into the mice. An analysis performed in 2 weeks after inoculation showed that the number of GFP+ fibroblasts in the tumor was, on average, 1–2 orders of magnitude higher for pPCNA than that for pCMV. The decreased proliferation of fibroblasts and silencing of transgene expression observed in vitro for pCMV fibroblasts results in the decreasing of proportion of GFP-positive fibroblasts in tumors.

The results obtained showed that:

1. Each of the fibroblast culture in this study had its own quantitative characteristics that were different from those of the others.
2. Nevertheless, we observed the following general qualitative properties: (a) the transgene expression and the fluorescence intensity of the reporter *GFP* gene under the control of pCMV decreased significantly with time in all the cultures studied; (b) these two parameters were most stable under pPCNA and pIGFBP2; (c) the expression of transgene under the control of pPCNA was more efficient than that under pIGFBP2.
3. Practically in all the cases studied, changes in the transcription level of transgene under pPCNA and pIGFBP2 correlated with changes in the transcription level of the *PCNA* and *IGFBP2* genes.
4. After transduction into fibroblasts, the cytotoxic effect of the *HSVtk*/GCV system only slightly depended on the promoter strength. However, during prolonged culture of the fibroblasts, the cytotoxic effect was decreased, which is especially characteristic of pCMV.
5. In vivo, the fibroblasts modified by a transgene under the control of pPCNA retained their ability to maintain expression of the transgene, in contrast to those modified with transgene under the control of pCMV.

Thus, the present work demonstrates that the ectopic activity of isolated promoters used for the expression of transgene in genetically modified fibroblasts correlates with the activity of native cellular promoters. It may mean that ectopic promoters at least partially retain their ability to interact with the remote regulation system of the gene controlled by a given promoter. However, this interesting finding requires further studies.

In addition, the data obtained in this study allow us to make the following practical recommendations:

The use of the CMV promoter for expression of transgenes in fibroblasts transduced with lentiviral vectors is not reasonable. The constitutive PCNA promoter is the most efficient for long-term expression of transgenes both in vitro and in vivo.

The hypersensitivity of fibroblasts to suicide gene therapy that was observed in our work may cause limitations in use of CAFs as a long-term source of toxins in tumors. We speculate that CAFs could be used better for the expression of other therapeutic genes, which is a subject for further research.

## 4. Materials and Methods

### 4.1. Promoters Used

The pCMV used was CMV Pr/Enh promoter containing an AseI/BglII fragment of the promoter of early cytomegalovirus genes from plasmid pEGFP-N1 (Clontech Laboratories (Takara Bio), Shiga, Japan) [40].

The pPCNA used was the promoter of the human *PCNA* gene. PCNA is one of the key participants in the replication process. In our study, we used a PCNA short promoter (389 bp, coordinates −241/+148 with respect to the transcription start site) that was earlier studied in our laboratory [29] and is considered a strong universal promoter for mammalian cells.

The pIGFBP2 used was the promoter of the human *IGFBP2* gene. For cloning and further study, we used a fragment of the IGFBP2 promoter with coordinates −635/−2 relative to the transcription start site. The promoter was amplified from the human genomic DNA using KAPA2G Robust DNA polymerases (Kapa Biosystems (Merck), Darmstadt, Germany) and primers IGFBP2-F634 and IGFBP2-R135+ (Table 3). The amplified DNA fragment was first cloned into apAL-TA vector (Evrogen, Moscow, Russia) and then re-cloned into a pGL3 Basic Vector (Promega, Madison, WI, USA). The pGL3IGFBP2 plasmid containing pIGFBP2 in the required orientation was isolated by restriction analysis and confirmed by DNA sequencing.

### 4.2. Viral Vector Plasmid and Virus Production

All lentiviral vectors were designed based on the modified self-inactivating lentiviral vector pLVPuro (kindly provided by Prof. V.V. Belousov, Institute of Bioorganic Chemistry, Russian Academy of Sciences, Moscow, Russia), which solely expresses the puromycin-resistance gene under the control of the PGK promoter. The modified pLVP (pLV-Polylinker) vector was obtained by cloning a polylinker (a duplex with multiple cloning sites obtained by annealing the primers LV_For_L and LV_Rev_L (Table 3)) into the SanDI restriction enzyme site. The pCMV-HSVtk-P2A-CopGFP, the pPCNA-HSVtk-P2A-CopGFP, the pCMV-EGFP-P2A-Luc2, and the pPCNA-EGFP-P2A-Luc2 cassettes were derived from previously obtained corresponding plasmids based on the pGL3 vector (Promega) and inserted into the prepared pLVP vector. The cassettes did not contain polyadenylation signal after the stop-codon and were in direct orientation relative to the LTR. The nucleotide sequences of the cassettes in all the plasmids were confirmed by sequencing. The promoter of the human *IGFBP2* gene was amplified from a pGL3pIGFBP2 plasmid by PCR using the primers IGFBP2-Mlu-R/IGFBP2-Sal-F containing restriction sites. The amplification product was digested with MluI and SalI restriction endonucleases (Thermo Fisher Scientific, Waltham, MA, USA) and cloned into a pLVP vector to replace the CMV-promoter sequence in the plasmid with the IGFBP2-promoter sequence. The nucleotide sequences of pCMV, pPCNA, and pIGFBP2 promoters as well as the *CopGFP* gene and the DNA sequence of the P2A peptide are given in Supplementary Information.

For virus production, cells of 293T cell line were transiently transfected with a viral vector plasmid along with a mixture of auxiliary plasmids (pMD.G and pCMV-dR8.91) using Lipofectamine 2000 (Invitrogen, Carlsbad, CA, USA) according to the manufacturer’s recommendations. After transfection, the cells were first incubated for 24 h at 37 °C, and then for 24 h at 32 °C prior to the collection of viral supernatants. The supernatant medium containing the lentivirus was then harvested, filtered, and stored at −70 °C as described earlier [41].

### 4.3. Cell Lines

Mouse embryonic fibroblast cell lines NIH/3T3 (ATCC^®^ CRL-1658™) and BALB/3T3 clone A31 (ATCC^®^ CCL-163™), mouse connective tissue fibroblast cell line L-929 (ATCC^®^CCL-1™), mouse colorectal carcinoma cell line CT26.WT (ATCC^®^ CRL-2638™), and 293T cell line (ATCC^®^ CRL-3216™) were obtained from the American Type Culture Collection (ATCC, Manassas, Virginia, USA).

A primary cell culture of human fibroblasts, IVP-15TS, was provided by the Vishnevsky Institute of Surgery (Moscow, Russia) [42].

NIH/3T3, BALB/3T3, and 293T cell lines and IVP-15TS fibroblasts were cultured in Dulbecco’s modified eagle’s medium (DMEM) supplemented with 10,000 U/mL penicillin, 10 mg/mL streptomycin, and 10% fetal bovine serum (FBS). L-929 and CT26.WT cell lines were cultured in RPMI 1640 medium supplemented with 10,000 U/mL penicillin, 10 mg/mL streptomycin, and 12.5% fetal bovine serum. The media and the supplements were purchased from Gibco (Thermo Fisher Scientific, Waltham, MA, USA). Cells were maintained in a humidified atmosphere at 5% CO_2_ and 37 °C.

The doubling time of the embryonic fibroblast cell line NIH/3T3 was 20–26 h (http://www.nih3t3.com/cell-culture-information/), and that of the cell line L-929 was 21–24 h (http://bioinformatics.hsanmartino.it/cldb/cl3158.html). There was no difference in cell proliferation of mouse cell lines after thawing.

The cell lines were passaged twice every week after the cells reached 90–100% confluency.

### 4.4. Lentiviral Transduction of Cell Lines and Primary Culture of Fibroblasts

In brief, the cells of the mouse fibroblast cell lines and primary culture of fibroblasts were seeded in 25 cm^2^ culture flasks at approximately 20–30% confluency and infected with lentiviral particles on the next day of seeding. For transduction, the supernatant was discarded first, and then an appropriate amount of each virus was added to the cultures. The cells were incubated with the viral supernatants at 37 °C in a humidified atmosphere at 5% CO_2_ for 4–5 h. Next, up to 5 mL of corresponding medium was added to the cells, and they were cultured for further 20 h. The successfully transduced cells were selected using growth medium containing 1.5 µg/mL (for NIH/3T3), 2.4 µg/mL (for BALB/3T3), 7.5 µg/mL (for L-929), or 0.5 µg/mL (for IVP-15TS) puromycin for 7–9 days. The number of GFP-expressing cells and the GFP fluorescence intensity in each sample were analyzed twice, 1 and 4–5 weeks (4 weeks for IVP-15TS, 5 weeks for NIH/3T3 and L-929) post transduction. In order to isolate total RNA or genomic DNA and evaluate the cytotoxicity of HSVtk/GCV suicide gene system, cells were harvested or seeded at the same time points after transduction (after 1 week as well as after 4–5 weeks of transduction). For inoculation in mice, cells were harvested after 3 weeks of puromycin selection and prepared as described below (see In Vivo Assay section).

### 4.5. Isolation of Nucleic Acids

To isolate total RNA or genomic DNA, cells were trypsinized and washed twice with phosphate-buffered saline (PBS). Then, the cell pellet was collected and stored at −70 °C prior to isolation of DNA and/or RNA.

Total RNA was isolated from 1 million cells using an RNeasy Mini Kit (Qiagen, Venlo, Netherlands) followed by treatment with DNAse RQ1 (Promega) according to the manufacturer’s protocol. Isolation of genomic DNA was performed using a High Pure PCR Template Preparation Kit for genomic DNA (Roche, Basel, Switzerland) according to the manufacturer’s protocol. The quality of RNA and DNA was analyzed by electrophoresis in a 1% agarose gel containing ethidium bromide. The amount of RNA and DNA was determined with a NanoDrop 2000 spectrophotometer (Thermo Fisher Scientific) at the absorption wavelength of 260 nm.

### 4.6. Evaluation of Lentivirus Integration

The integration of lentiviruses into the genomic DNA of the transduced mouse fibroblast cell lines was evaluated by qPCR. Genomic DNA of the transduced cell lines was isolated and used as a template. Each qPCR reaction mixture (Evrogen) contained 20 ng of the template DNA, SYBR Green I dye, and the following primer pairs: Arf1-ForE3/Arf1-RevE5 (per genomic site) and comLV1 for/comLV1 rev (per lentiviral site, Table 3). The data were processed to determine the amount of lentivirus integration, where *Arf1* was used as a reference gene.

### 4.7. Transcription Analysis by qPCR

The transcription level of the transgene controlled by various promoters as well as the constitutive transcription level of the *PCNA* and *the IGFBP2* genes in the transduced NIH/3T3 and L-929 cell lines and IVP-15TS primary culture was evaluated by qPCR using a qPCRmix-HS SYBR reaction mixture (Evrogen). The first cDNA strands were synthesized using hexanucleotide primers and Mint reverse transcriptase (Evrogen) according to the manufacturer’s protocol. For this purpose, total RNA was isolated from the cell pellets of the corresponding cell lines collected after 1 or 4–5 weeks after transduction. A universal primer pair, HSVtk-f90/GFP-R59, was used to determine the transcription level of the transgenes. The IGFBP2-forE4/IGFBP2-revE4.1 and the PCNAfor/PCNA-rev primer pairs were used to determine the transcription level of the *PCNA* or the *IGFBP2* genes in IVP-15TS fibroblasts. Additionally, mIGFBP2-For/mIGFBP2-Rev and mPCNA-for/mPCNA-rev primer pairs were used for the mouse fibroblast cell lines (NIH/3T3, L-929) (Table 3). Data were normalized relative to the geometric mean of the transcription level of the *18S RNA*, the *GPI*, and the *EEF1A1* genes for the primary culture of human fibroblasts and relative to the geometric mean of the transcription level of the *m18S RNA*, the *Psmb7*, and the *Rab1b* genes for the mouse fibroblast cell lines. Statistical processing of the data was performed using Excel, LinRegPCR, and LC480Conversion.

### 4.8. Evaluation of the GFP Reporter Expression

After 1 or 4–5 weeks of transduction, NIH/3T3, L-929, and IVP-15TS cells were collected for cytofluorimetric analysis to determine the intensity of GFP fluorescence and number of GFP+ cells. To evaluate the GFP expression, we took the value obtained in the green channel FL1 of a FACS cytometer (Becton Dickinson, Franklin Lakes, NJ, USA) and normalized it to the control (native cells). About 20000 gated events were selected for evaluation. For data processing, the program Flowing Software 2.5.1 was used.

### 4.9. Cell Viability Analysis (MTS Test)

To characterize the effect of ganciclovir on the transduced cell lines NIH/3T3 and L-929 and IVP-15TS fibroblasts, the cells were seeded at 4000 cells/well in a 96-well plate and cultured for 24 h at 37 °C and 5% CO_2_ after 1 week and 4–5 weeks of transduction. The growth medium was changed with medium containing ganciclovir (Sigma-Aldrich, St. Louis, MI, USA) at various concentrations including 0, 2, 12.5, 50, and 200 μM (for the human fibroblasts primary culture) or 0, 0.5, 2, 12.5, 50, and 200 μM (for the mouse fibroblast cell lines). The medium was changed on days 1 and 4 of culturing, and MTS test was performed on day 6 to assess cell viability. Approximately 20 μL of the MTS reagent CellTiter 96 AQueous One Solution (Promega, Madison, WI, USA) was mixed with 100 μL of appropriate medium and added to each well of the 96-well plate. The cells were incubated for 1 h at 37 °C and 5% CO_2_, and then their optical density was measured with an automatic table spectrophotometer (Benchmark™ Plus Microplate Reader, Bio-Rad, Hercules, CA, USA) at 590 nm. The data were processed with the Excel program. In all cases, non-transduced cells were used as control to assess the possible toxicity of ganciclovir (not observed).

### 4.10. Determination of Relative Cell Proliferation Rate

To determine changes in the rate of cell proliferation after transformation of cells with different transgenes, we performed MTS analysis. During the analysis, MTS tetrazolium compound is converted into its colored derivative in live cells. Thus, a decrease in the color intensity corresponds to a decrease in the number of live cells. To carry out the MTS test, cells were seeded into 96-well plates (4000 cells/well) and cultured for 6 days, after which the MTS reagent was added.

### 4.11. In Vivo Assay

The Balb/c mice (6–8 weeks) were obtained from the Pushchino Animal Breeding Facility (branch of the Shemyakin-Ovchinnikov Institute of Bioorganic Chemistry, Russian Academy of Sciences). The studies using mice were reviewed and approved by the *Shemyakin-Ovchinnikov* Institute of bioorganic chemistry Institutional Animal Care and Use Committee (IACUC, protocol No. 244). All animal manipulations were performed according to the recommendations of the European Convention for the Protection of Vertebrate Animals Used for Experimental and Other Scientific Purposes, Council of Europe (ETS 123). For tumor generation, 100 μL of PBS solution containing 3 × 10^5^ modified or native BALB/3T3 cells and 1 × 10^5^ CT26.WT cancer cells were injected subcutaneously into both flanks of the lower part of the mouse body. This was done in accordance with the 3R principles to reduce the number of animals in the experiment. After 14 days, when the tumors reached the size of 50–200 mm^3^, they were isolated and used for preparation of cell suspensions.

### 4.12. Preparation of Cell Suspensions from Tumors and Protein Assay

The tumors were washed with PBS, grinded with scissors in DMEM/F12 medium containing 1% FBS, a mixture of antibiotics, and 1 mg/mL Collagenase D from Clostridium histolyticum and incubated at 37 °C in a CO_2_ incubator for 3 h. The resulting mixture was resuspended, passed through a 70 μm cell strainer, centrifuged at 160 g for 5 min at 4 °C, and washed twice in PBS. The cells were then resuspended in FACS buffer. A cytofluorimetric analysis was performed to determine the intensity of GFP fluorescence and the number of GFP+ cells as described above.

The cell suspension was lysed by adding 150 μL of 1 × PLB (Passive Lysis Buffer, Promega) buffer to 200,000 cells. The aliquots of the resulting lysates were used to determine the luciferase activity and amount of protein by the Bradford method.

### 4.13. Luciferase and Bradford Protein Assays

To measure the amount of protein, the samples were mixed with a dye reagent in the ratio of 4:1 and incubated at room temperature for 10 min with constant stirring. The absorbance was measured with the Benchmark™ Plus Microplate Reader (Bio-Rad) at 595 nm. The activity of firefly luciferase 2 in cell extracts was measured using a Dual-Luciferase Reporter Assay System (Promega) and GENios Pro (Tecan, Mannedorf, Switzerland) luminometer. The luciferase activity was normalized to that of 1 mg of protein. Calculations were made using Excel program.

## Figures and Tables

**Table 1:** Values of the 50% inhibitory concentration (IC_50_, µM) of ganciclovir in transduced cells that contain the transgene *HSVtk-CopGFP* under the control of pCMV, pPCNA, and pIGFBP2. Cell viability was measured by MTS test.

Promoter	IVP-15TS		NIH/3T3		L-929	
	In 1 Week after Transduction	In 4 Weeks after Transduction	In 1 Week after Transduction	In 5 Weeks after Transduction	In 1 Week after Transduction	In 5 Weeks after Transduction
pCMV	10.37 ± 0.55	N/A	0.13 ± 0.05	0.10 ± 0.01	0.10 ± 0.02	0.73 ± 0.03
pPCNA	9.03 ± 0.19	24.35 ± 0.53	0.16 ± 0.01	0.09 ± 0.01	0.06 ± 0.01	0.07 ± 0.01
pIGFBP2	11.37 ± 0.70	72.92 ± 1.59	0.09 ± 0.02	0.15 ± 0.02	0.13 ± 0.02	0.09 ± 0.01

The analysis was performed after 1 and 4 weeks of transduction for the primary culture (IVP-15TS) and after 1 and 5 weeks of transduction for linear fibroblasts NIH/3T3 and L-929. The measurements were carried out in three technical replicates for each sample and are represented as mean ± s.e.m. N/A: no data obtained because ganciclovir at the concentrations used decreased the survival rate of the cells by 50%.

**Table 2:** Analysis of the GFP+ cells content and luciferase activity in a suspension of tumor cells containing co-cultures of cancer line CT26.WT and various BALB/3T3 fibroblasts.

Modification	Relative 10^3^ RLU/ mg *	Percentage of GFP+ Cells	Fluorescence Intensity	
			X GeoMean	X Median
pPCNA-GFP-P2A-Luc2 n1	1428.3	4.08	42	43
pPCNA-GFP-P2A-Luc2 n2	1288.7	3.36	41	45
pPCNA-GFP-P2A-Luc2 n3	638.8	1.19	40	44
pPCNA-GFP-P2A-Luc2 n4	82.3	0.16	38	39
pCMV-GFP-P2A-Luc2 n1	56.9	0.03	16	14
pCMV-GFP-P2A-Luc2 n2	379.0	0.29	14	11
pCMV-GFP-P2A-Luc2 n3	68.9	0.11	8	7
Control n1	4.4	0	-	-
Control n2	1.1	0	-	-

* Relative Light Units per milligram of protein.

**Table 3:** Primers used in the experiments.

Primers Used in qPCR			
Gene	Species Affiliation	Primer	Primer Sequence
Transgene (*HSVtk/GFP*)	-	HSVtk-f90	5′-ATGGTCCAGACCCACGTCA-3′
		GFP-R59	5′-GTGCCGGTGATGCGGCACT-3′
*IGFBP2*	Human	IGFBP2-forE4	5′-AGATGTCTCTGAACGGGCAG-3′
		IGFBP2-revE4.1	5′-AAGAGATGACACTCGGGGTC-3′
	murine	mIGFBP2-For	5′-AGATGTCTCTGAACGGACAG-3′
		mIGFBP2-Rev	5′-GAGATGGCACTCGGGGTC-3′
*PCNA*	Human	PCNA-for	5′-GCTCCATCCTCAAGAAGGTGT-3′
		PCNA-rev	5′-GCAAATTCACCAGAAGGCATC-3′
	murine	mPCNA-for	5′-GCTCCATCCTGAAGAAGGTG-3′
		mPCNA-rev	5′-CAAATTCACCCGACGGCATC-3′
*18S RNA*	Human	18S-for	5′-CGCGGTTCTATTTTGTTGGT-3′
		18S-rev	5′-ATGCCAGAGTCTCGTTCGTT-3′
	murine	m18S-for	5′-TGCAATTATTCCCCATGAACG-3′
		m18S-rev	5′-GCCTCACTAAACCATCCAATC-3′
*GPI*	Human	GPI-forE2	5′-GACCGCTTCAACCACTTCAG-3′
		GPI-revE3	5′-CTCCGTCACCAGGTTCTTG-3′
*EEF1A1*	Human	EEF1A1-forE1	5′-GACACGTAGATTCGGGCAAG-3′
		EEF1A1-revE2	5′-GATACCACGTTCACGCTCAG-3′
*Psmb7*	murine	Psmb7-forE1	5′-GCGGCTGTGTCGGTGTTTC-3′
		Psmb7-revE3	5′-CCTTCAGTTGCTCTCGTGTC-3′
*Rab1b*	murine	Rab1b-forE4	5′-CTGGTCAGGAGCGGTTCAG-3′
		Rab1b-revE5	5′-TCTTGGTGGTGAGGTCACTC-3′
**Primers Used for Obtaining Lentiviral Constructs**			
**Primer**		**Primer Sequence**	
LV_For_L		5′-CCGAGCGCTGTCGACGATATCACGCGTGG-3′	
LV_Rev_L		5′-GTCCCACGCGTGATATCGTCGACAGCGC-3′	
**Primers Used to Determine Viral Integration**			
comLV1 for		5′-CCACCAAGGCAAAGAGAAGA-3′	
comLV1 rev		5′-CTCCCAAGAACCCAAGGAAC-3′	
Arf1-ForE3		5′-AGCTTCACCGTGTGGGATG-3′	
Arf1-RevE5		5′-AGTTCCTGTGGCGTAGAGAG-3′	
**Additional Primers**			
T7		5′-TAATACGACTCACTATAGGG-3′	
Sp6		5′-CGATTTAGGTGACACTATAG-3′	
IGFBP2-Mlu-R		5′-ATTACGCGTCTGGCGGTCGGCAGCGC-3′	
IGFBP2-Sal-F		5′-TAAGTCGACTAGACGGGTCTGAAACTC-3′	
IGFBP2-F634		5′-GGCAGGTTTTGCGGGGCAC-3′	
IGFBP2-R135+		5′-CAGCCCACTCTCGGCAGCAT-3′	

## Supplementary Materials

Supplementary materials can be found at https://www.mdpi.com/1422-0067/21/17/6098/s1.
