# Supplementary material for: Searching for Promoters to Drive Stable and Long-Term Transgene Expression in Fibroblasts for Syngeneic Mouse Tumor Models
Source: Int J Mol Sci. 2020 Aug 24;21(17):6098. doi: 10.3390/ijms21176098 (PMC7504129; doi:10.3390/ijms21176098)
Supplement: Supplementary file 1 [file ijms-21-06098-s001.pdf]

Supplementary Figure S1. Evaluation of lentivirus integration. Analysis of the transgene abundance in stably transduced fibroblasts one or five weeks after transduction. The transcription level of the transgene was under the control of pCMV, pPCNA, or IGFBP2 promoters. The experiments were performed in triplicate for each sample. The height of each box represents the mean average of a sample, while associated error bars represent ± s.e.m.

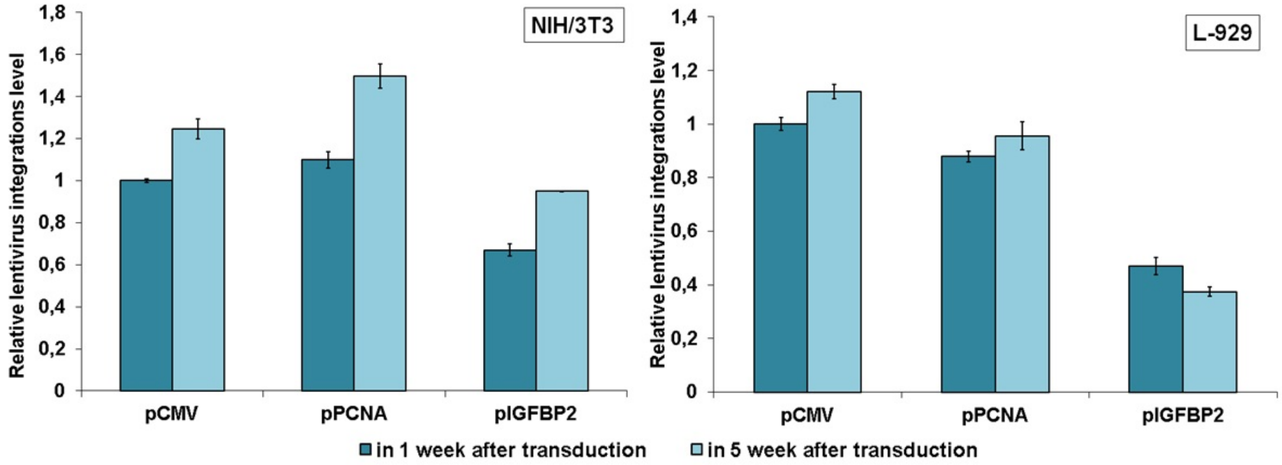

Nucleotide sequences of the DNA elements used for this study:

> pCMV (581 bp)

taatagtaatcaattacggggtcattagttcatagcccatatatggagttccgcgttacataac
ttacggtaaatggcccgcctggctgaccgcccaacgacccccgcccattgacgtcaataatgac
gtatgttcccatagtaacgccaatagggactttccattgacgtcaatgggtggagtatttacgg
taaactgcccacttggcagtacatcaagtgtatcatatgccaagtacgccccctattgacgtca
atgacggtaaatggcccgcctggcattatgcccagtacatgaccttatgggactttcctacttg
gcagtacatctacgtattagtcatcgctattaccatggtgatgcggttttggcagtacatcaat
gggcgtggatagcggtttgactcacggggatttccaagtctccaccccattgacgtcaatggga
gtttgttttggcaccaaaatcaacgggactttccaaaatgtcgtaacaactccgccccattgac
gcaaatgggcggtaggcgtgtacggtgggaggtctatataagcagagctggtttagtgaaccgt
cagat

> pPCNA (389 bp)

tctccacatatgcccggacttgttctgcggccgggttcaggagtcaaagaggcggggagacctg
cgcgacgctgccccgccctgcgcccgcttcctccaatgtatgctctagggggcgggcctcgcgg
ggagcatggacacgattggccctaaagtcttccccgcaaggccgtgggctggacagcgtggtga
cgtcgcaacgcggcgcagggtgagagcgcgcgcttgcggacgcggcggcattaaacggttgcag
gcgtagcagagtggtcgttgtctttctaggtctcagccggtcgtcgcgacgttcgcccgctcgc

tctgaggctcctgaagccgaaaccagctagactttcctccttcccgcctgcctgtagcggcgtt
gttgc

> pIGFBP2 (633 bp)

ctagacgggtctgaaactccgcaggacccacccaacaagaagtcattgttccaagccacgtgtc
agtggtgggtgataccccaggatggaaggagttggtatgagccgactgaaatctacttgaaggt
caaaacggagccttatgtcttttgtgttccccagcggttagcccagtgccggccacaggggaag
cgcgcaaacgaagtcctcgcgaactgaactgagagcagacaaaagcacgcgctcttctccaccg
ccacgccggtcctacccaaacccgcgagttatccgtattctccttcaggagtcatagtcaggcc
agaagagtgcggagggacggggcccgggaagagcagggaacccccagagcccgcagccaacgcg
gaggtgggcgagcgggcgtgcgcgcactcacttgccggcgcgagggagtgtcgggggggaaggg
agtggtctccaaaaggggggaggggagaaggcaggggggcggggagaagccggccctttaggaccc
ggctgcggcggcgagggaggaggaagaagcggaggaggcggctcccgcgctcgcagggccgtgc
cacctgcccgcccgcccgctcgctcgctcgcccgccgcgccgcgctgccgaccgcca

> *CopGFP* gene (CopGreen2) (741 bp)

atggagagcgacgagagcggcctgcccgccatggagatcgagtgccgcatcaccggcaccctga
acggcgtggagttcgagctggtgggcggcggagagggcacccccaagcagggccgcatgaccaa
caagatgaagagcaccaaaggcgccctgaccttcagcccctacctgctgagccacgtgatgggc
tacggcttctaccacttcggcacctaccccagcggctacgagaaccccttcctgcacgccatca
acaacggcggctacaccaacacccgcatcgagaagtacgaggacggcggcgtgctgcacgtgag
cttcagctaccgctacgaggccggccgcgtgatcggcgacttcaaggtggtgggcaccggcttc
cccgaggacagcgtgatcttcaccgacaagatcatccgcagcaacgccaccgtggagcacctgc
accccatgggcgataacgtgctggtgggcagcttcgcccgcaccttcagcctgcgcgacggcgg
ctactacagcttcgtggtggacagccacatgcacttcaagagcgccatccaccccagcatcctg
cagaacgggggccccatgttcgccttccgccgcgtggaggagctgcacagcaacaccgagctgg
gcatcgtggagtaccagcacgccttcaagacccccatcgccttcgccagatccagagcccaggc
cagcaactccgccgtggatggcacagccggaccgtaa

> P2A-peptide (69 bp)

accggttccggagccacgaacttctctctgctgaagcaagcaggagacgtggaggagaatcccg
ggcct
